# The Role of Genomic Islands in the Pathogenicity and Evolution of Plant-Pathogenic Gammaproteobacteria

**DOI:** 10.3390/microorganisms13081803

**Published:** 2025-08-01

**Authors:** Yuta Watanabe, Yasuhiro Ishiga, Nanami Sakata

**Affiliations:** 1 The Graduate School of Environmental, Life, Natural Science and Technology, Okayama University, 1-1-1 Tsushima-naka, Kita-ku, Okayama 700-8530, Japan; 2 Faculty of Life and Environmental Sciences, University of Tsukuba, 1-1-1 Tennodai, Tsukuba 305-8572, Japan; 3 Faculty of Agriculture, Okayama University, 1-1-1 Tsushima-naka, Kita-ku, Okayama 700-8530, Japan

**Keywords:** integrative and conjugative elements, horizontal gene transfer, virulence factors, *Pseudomonas syringae*, backbone genes, cargo genes, ICE*clc* regulatory network

## Abstract

Genomic islands (GIs) including integrative and conjugative elements (ICEs), prophages, and integrative plasmids are central drivers of horizontal gene transfer in bacterial plant pathogens. These elements often carry cargo genes encoding virulence factors, antibiotic and metal resistance determinants, and metabolic functions that enhance environmental adaptability. In plant-pathogenic species such as *Pseudomonas syringae*, GIs contribute to host specificity, immune evasion, and the emergence of novel pathogenic variants. ICE*clc* and its homologs represent integrative and mobilizable elements whose tightly regulated excision and transfer are driven by a specialized transcriptional cascade, while ICEs in *P. syringae* highlight the ecological impact of cargo genes on pathogen virulence and fitness. Pathogenicity islands further modulate virulence gene expression in response to in planta stimuli. Beyond *P. syringae*, GIs in genera such as *Erwinia*, *Pectobacterium*, and *Ralstonia* underpin critical traits like toxin biosynthesis, secretion system acquisition, and topoisomerase-mediated stability. Leveraging high-throughput genomics and structural biology will be essential to dissect GI regulation and develop targeted interventions to curb disease spread. This review synthesizes the current understanding of GIs in plant-pathogenic gammaproteobacteria and outlines future research priorities for translating mechanistic insights into sustainable disease control strategies.

## 1. Introduction

Plant bacterial diseases pose a significant global challenge, driving substantial crop losses and undermining food security [1]. The spread of these diseases is fueled by international trade, climate change, and the emergence of novel pathogens, while rapid pathogen evolution often overcomes host resistance [2,3]. Traditional control measures, such as copper-based bactericides, are losing efficacy and raise environmental concerns [4].

A key driver of bacterial adaptability is horizontal gene transfer (HGT), particularly via genomic islands (GIs). These large, mobile DNA segments, typically 10 to 200 kb, harbor genes that confer selective advantages, including virulence factors, antimicrobial resistance, and stress tolerance [5,6]. GIs are distinguished from the core genome by atypical GC content, integration near tRNA loci, and flanking direct repeats or integrase sites, reflecting their diverse origins and transfer mechanisms [7,8].

Among GIs, pathogenicity islands merit particular attention: they encode secretion systems, toxins, and enzymes that facilitate host invasion and immune evasion [9,10]. The dynamic acquisition and loss of GIs enable bacterial pathogens to swiftly adapt to environmental pressures, such as antimicrobial treatments and host defenses, complicating disease management [11,12,13].

This review synthesizes current knowledge on the structure, transfer, and evolutionary impact of GIs in plant-pathogenic gammaproteobacteria. We highlight their roles in virulence and resistance, discuss methodological advances in GI detection and classification, and consider their implications for developing sustainable disease control strategies.

## 2. Structure and Transfer Mechanisms of ICEs

The category of GIs includes integrative plasmids, prophages, and integrative and conjugative elements (ICEs). ICEs, in particular, are self-transmissible via conjugation and are highly conserved across a broad range of bacterial species [14,15]. Some ICEs have been identified as pathogenicity islands, antibiotic resistance islands, or catabolic islands in pathogenic and environmental bacteria. Considerable research has focused on understanding how ICEs influence bacterial phenotypes and their mechanisms of transfer. Given that ICEs carry various backbone genes (core gene, functional module) essential for mobilization, they are classified into distinct families based on integrase (Int) similarity and on core structure synteny [16]. Several representative ICE families, including ICE*Bs1* (*Bacillus*), Tn*916* (*Escherichia*), ICE*SXT* (*Vibrio*), CTn*DOT*, ICE*clc* (*Pseudomonas*), and ICE*MISymR7A* (*Azorhizobium*), have been identified, although their transfer mechanisms are still being actively studied.

**Figure 1:**
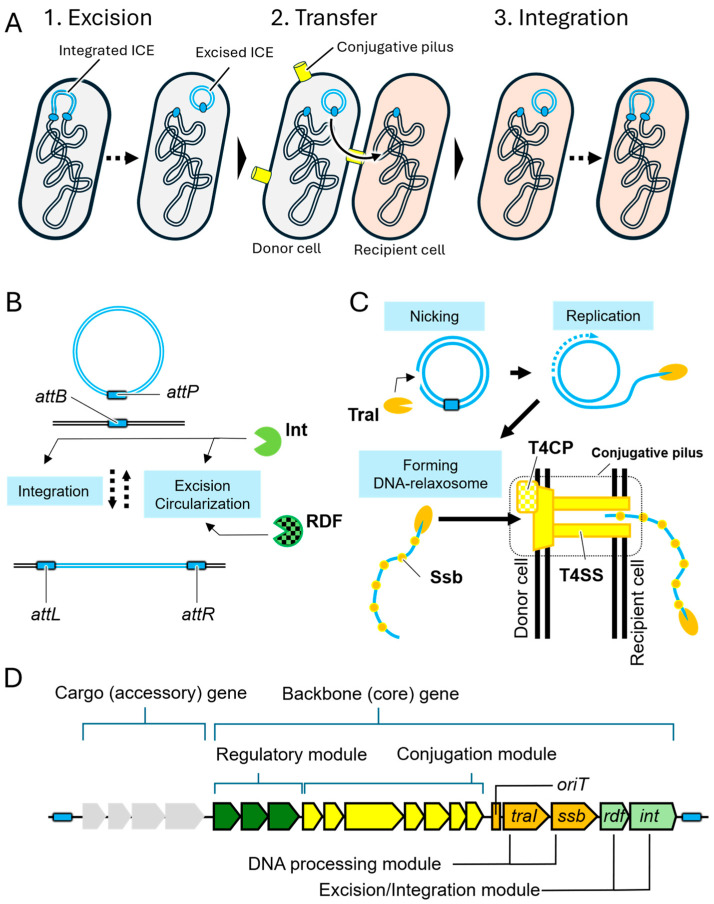
(**A**) Schematic overview of the transfer process of integrative and conjugative elements (ICEs). (**B**) The excision of ICEs (blue double lines) from the host chromosome (black double lines)**.** This reaction is proceeded by Integrase (Int, light green three-quarter circle) and recombination direction factors (RDFs, light green three-quarter circle with grid). (**C**) The transfer and integration of excised ICEs into recipient cells (light orange) via a conjugative pilus (yellow cylinder). By the action of TraI (orange three-quarter flat circle) and Ssb (single strand DNA binding protein, orange dot), excised ICE is modified and delivered to conjugative pilus consisting of type IV secretion system (T4SS, yellow object) and type IV coupling protein (T4CP, yellow grid object). (**D**) The gene composition of ICEs. ICE encode cargo genes (gray pentagon) and backbone genes including regulatory module (green pentagon), conjugation module (yellow pentagon), DNA processing module (orange pentagon), and excision/integration module (light green pentagon).

The reception and transfer of ICEs occurs in a stepwise process consisting of three primary stages: (1) excision, (2) DNA transfer, and (3) re-integration (Figure 1A, reviewed in [14,15,17]). Initially, the chromosomally integrated ICE is excised through the activity of an integrase (Int) (Figure 1B). In several ICE systems, integrase function is often supported by accessory proteins, recombination directionality factors (RDFs), including excisionase [18,19,20]. Excision typically occurs at specific short direct-repeat sequences known as *attL* and *attR* (Figure 1B), resulting in the formation of a double-stranded circular ICE and the restoration of the *attB* site on the host chromosome and the *attP* site on the excised element. Following excision, the circular ICE undergoes processing by specific DNA processing enzymes to prepare for transfer to recipient cells (Figure 1C). At this stage, the ICE is nicked at the origin of transfer (*oriT*) site by DNA relaxase, such as TraI, generating a linear single-stranded DNA (ssDNA). In some systems, the ICE replicates prior to transfer, enabling the mobilization of multiple copies. The relaxase TraI remains covalently attached to the 5′ end of the ssDNA, forming a relaxosome. This complex is coated with single-stranded DNA binding proteins (Ssb) and is guided to the conjugative machinery, comprising the type IV coupling protein (T4CP) and the type IV secretion system (T4SS). In the recipient cell; the transferred ssDNA is circularized and converted into double-stranded DNA, after which it is integrated into the host genome via the action of integrase. Successful ICE transfer requires four essential genetic components involved in mobilization: *int*, *oriT*, *ssb*, and *traI* (Figure 1D). In addition to these genes, other backbone genes related to DNA secretion and regulatory functions are often conserved across ICE families. These conserved components can be functionally grouped into four modular categories: excision/integration, DNA processing, conjugation, and regulation [14,21] (Figure 1D).

Although the repertoire of backbone genes varies among ICE families, this review focuses on ICE*clc* and its homologous elements, which are among the best-characterized ICEs and are frequently associated with virulence in plant-pathogenic bacteria. ICE*clc* is a 103 kb genomic element originally identified in *Pseudomonas knackumusii* B13, a bacterium isolated from sewage [22]. ICE*clc* is self-transmittable and carries cargo genes involved in the degradation of 3-chlorobenzoic acid and aminophenol, conferring ecological fitness to the host [23]. Putative homologs of ICE*clc* are broadly conserved in β- and γ-proteobacteria, including several animal and plant pathogens [24]. In *Pseudomonas syringae*, four function ICEs have been reported, each contributing to either pathogenicity or environmental adaptability [25,26,27,28]. Additionally, several homologous ICEs, collectively referred to as PsICE, have been identified in *P. syringae* through comparative genomic analysis [29]. A comparative study identified approximately 30 genes that are highly conserved across ICE*clc*, SPI-7, ICE*hin1056*, ICE*Ye1*, ICE*Xcc1*, and PAPI-1, suggesting a shared backbone gene set across diverse ICE families [24]. While the precise function of these conserved genes remained elusive for many years, recent studies using ICE*clc* as a model have begun to elucidate their molecular roles, shedding light on the fundamental mechanisms of ICE-mediated mobility and adaptation. 

## 3. Specific Mechanisms of ICEclc for Excision and Transfer

**Figure 2:**
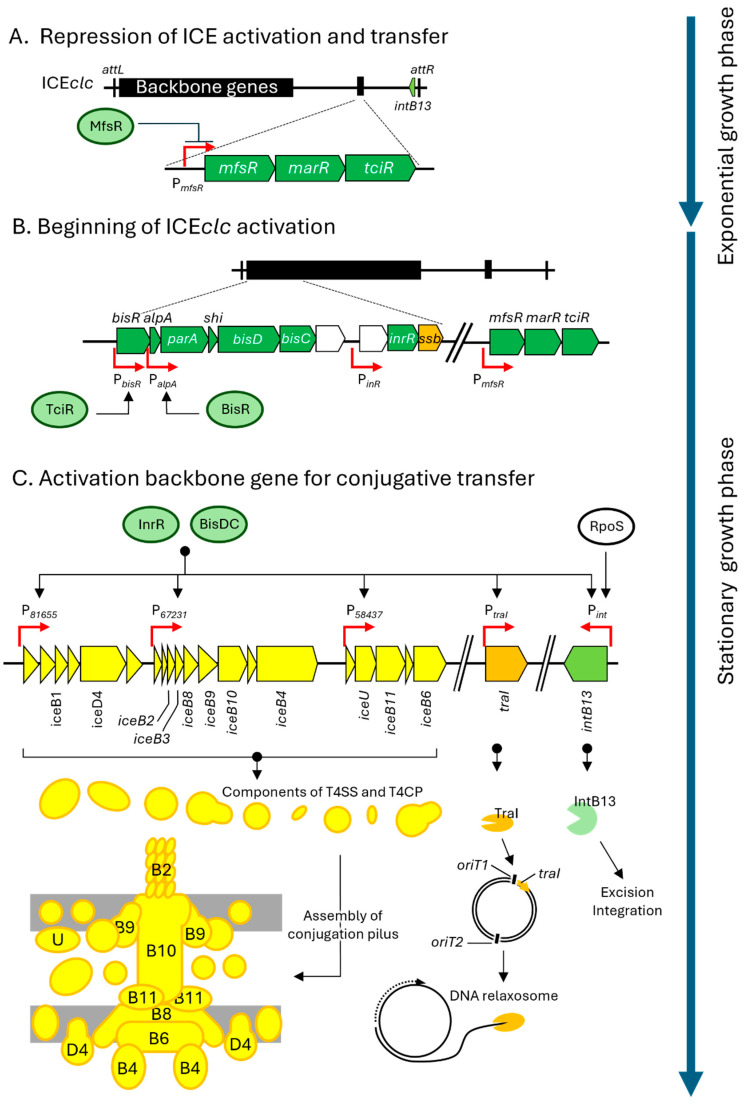

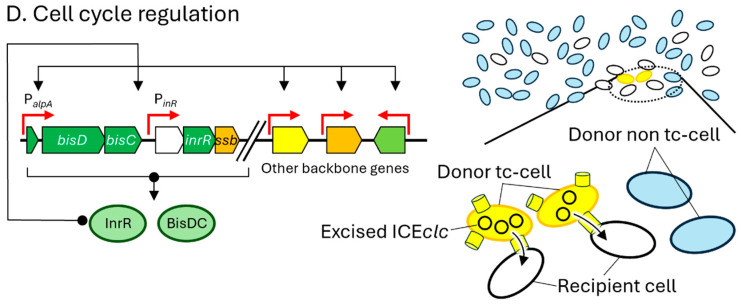
Regulatory cascade of ICE*clc* from excision to transfer. The pentagon objects in the figure are the same as those described in the legend of Figure 1. (**A**) ICE*clc* (black) remains integrated within the host chromosome and MfsR (green eclipse) interact to the *mfsR* promoter (P*_mfsR_*, red arrow) repressed its excision. (**B**) De-repression of *mfsR* and activation of *tciR* initiate ICE*clc* activation. (**C**) The BisDC complex (green eclipse), along with InrR (green eclipse), activates multiple backbone gene expressions. Their translation products play a role in conjugative pilus assembly and DNA processing, respectively. (**D**) The bistable differentiation into transfer-competent (tc) cells (yellow ellipse) and non-tc cells (blue ellipse) is maintained by a positive feedback loop involving BisDC and InrR.

ICE*clc* is typically integrated into the bacterial chromosome and remains transcriptionally silent under standard growth conditions. This inactive state is maintained by a regulatory gene cluster located on ICE*clc*, comprising *mfsR*, *marR*, and *tciR*, which together form a single transcriptional unit [30] (Figure 2A). Within this unit, TciR, a LysR-type transcriptional factor, serves as a positive activator of ICE*clc* backbone gene expression. In contrast, MfsR, a TetR-type repressor, negatively regulates *tciR* transcription, thereby maintaining the silenced state of ICE*clc*.

Under specific environmental conditions, particularly during stationary-phase growth with 3-chlorobenzoate as the sole carbon source, ICE*clc* becomes transcriptionally active in approximately 5% of the bacterial population [31]. This activation begins with the de-repression of *tciR*, allowing TciR to initiate a hierarchical regulatory cascade (Figure 2B). TciR first induces the expression of *bisR*, which encodes a putative transcription activator (Figure 2B). BisR, in turn, activates the *alpA* promoter (P*alpA*), leading to the expression of downstream genes, including transcription factors *bisD*, *bisC*, and *inrR* [32]. The BisD and BisC proteins form a heteromeric complex (BisDC), which, together with InrR, activate the promoters of backbone ICE*clc* genes. This tightly controlled regulatory circuit governs two major phenotypic outcomes: the conjugation pilus assembly and the subsequent transfer of ICE DNA [33].

The first step in ICE*clc* transfer is the assembly of the conjugation pilus. ICE*clc* encodes approximately 20 genes involved in pilus formation. These *ice* genes, are homologous to the *tra*/*vir* genes found in the Ti-plasmid of *Agrobacterium* and the F plasmid, indicating that ICE*clc* employs T4SS for DNA transfer [34]. Among these, 11 *ice* genes, along with three additional genes, have been identified as essential for T4SS-mediated conjugative transfer [34].

The second step is the execution of DNA transfer. This process is regulated by the transcriptional activators BisDC and InrR, which induce the expression of *traI* and *intB13* (Figure 2C). TraI is responsible for the site-specific nicking and rejoining at *oriT1*, one of the two *oriT* sites on ICE*clc* [35]. As described in Section 2, the excised and nicked ICE is subsequently recruited to the conjugation pilus via the action of TraI, which is essential for ICE*clc* mobilization (Figure 1C). Additionally, IntB13 is a tyrosine site-specific recombinase that mediates site-specific recombination between the *attL* and *attR* sites flanking ICE*clc*, each marked by 18 bp direct repeats. This recombination event results in the excision of ICE*clc* from the chromosome [36]. The expression of *intB13* is not only regulated by InrR and BisDC complex but also the stationary-phase sigma factor RNA polymerase, sigma S (RpoS) (Figure 2C). RpoS binds to the promoter region (P*_int_*) of *intB13* and promotes its transcription [37]. Although many ICEs and prophages employ RDFs, such as excisionases for excision, no such RDFs have yet been identified in ICE*clc* or other *Pseudomonas* ICEs.

Thus, BisDC and InrR activate several backbone genes required for ICE transfer. Notably, *bisDC* and *inrR* also positively regulate their own expression, forming a feedback loop that amplifies the transcriptional response and commits a subpopulation of cells to become transfer-competent (tc) cells [38] (Figure 2D). During the stationary phase, approximately 3–5% of the population differentiates into tc cells, which can efficiently mediate ICE transfer to recipient cells. Tc cells lose the ability to proliferate, but because they constitute a small fraction of the population, this cost does not impact overall population survival. The positive feedback loop involving BisDC and InrR maintains bistability in the population by ensuring a stable ratio between tc cells and non-tc cells (Figure 2D).

In summary, ICE*clc* contains 24 functional backbone genes. A total of 8 of these function as regulatory modules: *mfsR*, *tciR*, *bisR*, *alpA*, *parA*, *bisDC*, and *inrR*. Two genes, *traI* and *intB13*, form integration/excision and DNA processing modules, respectively. The remaining 14 genes encode components of the conjugative pilus machinery, responsible for DNA secretion.

## 4. Classification of ICE*clc* and Its Homologous

A systemic comparison of ICE*clc* and its homologous sequences across *Proteobacteia* has led to the identification of approximately 30 putative backbone genes, collectively referred to as the “hypothetical ICE” [24]. While many of these backbone genes are conserved among various ICE, notable structural and functional differences exist. Here, we summarize the characteristics of backbone genes in six well-characterized ICEs: ICE*clc*, found in *P. knackumusii* B13; PsICE, referred to as *P. syringae* ICE; PAPI-1, found in *P. aeruginosa*; ICE*hin1056*, found in *H. influenzae*; SPI-7, found in *S. enterica*; and ICE*Ye1*, found in *Y. enterocolitica*. Based on these comparisons, we propose a refined classification system for ICE families.

**Figure 3:**
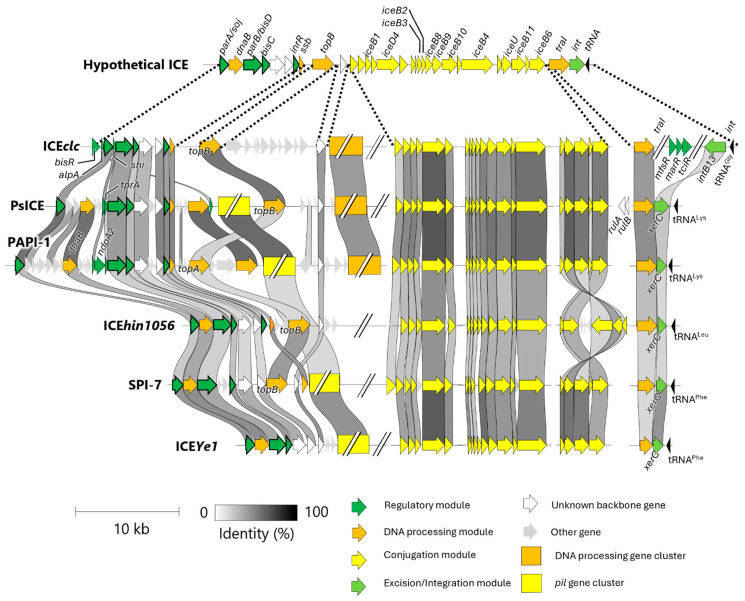
Backbone gene structures of ICE*clc* and five homologous ICEs. This comparative analysis is based on the conserved gene organization of ICE*clc* and five homologous integrative and conjugative elements (ICEs), as described by Mohd-Zain et al. [24]. Sequence similarity of backbone genes is represented by the intensity of shading across the ICEs. Among the backbone genes, regulatory, DNA processing, conjugation, excision/integration modules are shown as green arrow, orange arrow, yellow arrow, and light green arrows, respectively. Other unknown common backbone genes and other cargo genes shown as white arrow and gray arrow, respectively. DNA processing gene cluster and *pil* gene cluster are shown as orange rectangle and yellow rectangle, respectively.

Among these ICEs, four functional backbone genes, *parA*, *bisD*, *bisC*, and *inrR*, are conserved in all ICEs except ICE*Ye1* (Figure 3). Their conservation suggests that these elements likely regulate the transition from a non-tc state to a tc state during excision and mobilization similar to ICE*clc*. However, the full complement and organization of backbone genes vary among ICEs.

Three key differences in backbone gene structure distinguish these ICEs: (1) the presence or absence of a *pil* gene cluster; (2) the presence or absence of a conserved DNA processing gene cluster; and (3) the identity of topoisomerase and integrase genes (Figure 3). The conserved *pil* gene cluster, composed of at least 10 *pil* genes (yellow box in Figure 3), is present in PsICE, PAPI-1, ICE*Ye1*, and SPI-7. This region is syntenic with the *pil* gene cluster of the R64 conjugative plasmid, which enables liquid conjugation independent of the T4SS encoded by the ICE backbone genes [39,40].

A conserved DNA processing gene cluster upstream of *iceB1* containing a DNA helicase and several hypothetical genes (light green box in Figure 3) is shared among ICE*clc*, PAPI-1 and PsICE, although it is not important for ICE transfer and its function remains unknown [33]. Additionally, ICEs differ in their encoded topoisomerase and integrase genes. PAPI-1 encodes *topA*, whereas ICE*clc*, PsICE, ICE*hin1056*, and SPI-7 encode *topB*. Both genes encode type IA topoisomerase that resolve DNA entanglements [41]. In terms of integrase, ICE*clc* uniquely carries homologs of *intB13*, while the other ICEs encode homologs of *xerC*. Altough both IntB13 and XerC mediate ICE excision and integration [25,36,42], they differ in sequence and gene orientation. The biological significance of these structural differences is not yet fully understood, but they may offer useful criteria for ICE classification.

In the current ICE database (ICEberg), ICE families are defined primarily based on integrase amino acid sequence similarity. Accordingly, ICEs such as ICE*clc*, SPI-7, ICE*hin1056*, ICE*Ye1* and PAPI-1 are placed into distinct families. However, this method has limitations. For instance, *P. syringae* pv. *tabaci* harbors two ICEs, *Pta*GI-1 and *Pta*GI-2, that share nearly identical backbone gene structure but might be assigned to different families due to divergence in their integrase sequences [28]. This discrepancy suggests that integrase-based classification alone may not reliably capture evolutionary or functional relationships among ICEs.

To address these limitations, we advocate for a multi-criteria classification system, such as multilocus sequence analysis (MLSA), that incorporates not only integrase homology but also presence/absence of key gene clusters and overall backbone gene architecture. For example, HAI2, an ICE in *Pectobacterium atrosepticum* SCRI1043, shares structural features with SPI-7, including *pil* gene cluster, absence of the conserved DNA processing cluster, and identical topoisomerase and integrase genes, supporting its placement within SPI-7 family. Further, a putative ICE on *P. syringae* CC1557 (locus tags N018_RS12135-11815) lacks the *pil* gene cluster but contains the conserved DNA processing gene cluster and shares topoisomerase and integrase genes with ICE*clc*, aligning it more closely with the ICE*clc* family. These examples underscore the need for an integrative and structure-informed framework for ICE classification. As previously proposed by Bi et al. [16], such a framework should incorporate both backbone gene content and sequence homology to better reflect ICE diversity and evolutionary relationships.

## 5. Possible Mechanisms of PsICE Excision and Transfer

To understand the behavior and function of ICEs in *P. syringae* during host interactions, it is essential to investigate how *P. syringae* ICEs (PsICEs) are regulated and transferred through their backbone genes. However, the specific functions of PsICE backbone genes remain largely uncharacterized. Over the past two decades, the study of ICE*clc* has significantly advanced our understanding of ICE backbone gene function and mobilization. Since PsICEs share several homologs with ICE*clc*, its mechanistic insights provide a valuable framework to infer potential behaviors of PsICEs. However, differences in backbone gene architecture between PsICE and ICE*clc* (Figure 3) suggest divergence in regulatory mechanisms. In parallel, recent studies have revealed that the backbone genes of PsICEs are more highly conserved in PAPI-1, a well-characterized ICE in *P. aeruginosa*, than in ICE*clc* [42], suggesting that insights into backbone gene function may help clarify PsICE behavior. Therefore, comparing backbone gene structure among PsICE, ICE*clc* and PAPI-1 allows us to hypothesize functional attributes of PsICEs based on current models.

ICEs are generally excised from the chromosome by integrase, processed by TraI or Ssb, and subsequently transferred via the T4SS (Figure 1B). In ICE*clc*, *mfsR* and *tciR* repress ICE*clc* activation, and their de-repression leads *bisR* at the left end of the ICE*clc* (Figure 2A,B). This activates a transcriptional cascade, including *alpA*, *parA*, *bisD*, *bisC*, and *inrR*, which regulates the transition to tc cell-cycle state and modulate ICE*clc* mobilization (Figure 2B,C). Homologs of *ssb*, *traI*, *parA*, *bisD*, *bisC*, and *inrR* are conserved in PsICEs and other syntenic ICEs (Figure 3), suggesting that similar mobilization mechanisms may operate.

However, *mfsR*, *tciR*, *bisR*, and *alpA*, which are essential for transcriptional regulation in ICE*clc*, are absent in PsICEs and four other ICEs (Figure 3). Notably, these ICEs instead carry *parA* near the left end (Figure 3), implying an alternative regulatory strategy. In PAPI-1, the *parA* promoter is only active when the ICE is excised and circularized [41], suggesting that *parA* expression in PsICEs may also be excision-dependent. Additionally, the *rulAB* gene pair known to be induced by the SOS response [43] is conserved upstream of *traI* in PsICEs [29]. Given that the SOS responses activate ICEs in *Bacillus* and *Vibrio* [44], *rulAB* may function as an alternative regulatory module in PsICEs.

Three additional regulatory genes, *shi*, *tprA*, or *ndpA2*, are uniquely found in ICEs of *Pseudomonas* species (Figure 3). The *shi* located upstream of *bisC* in ICE*clc*, PsICE, and PAPI-1 arrests cell division and impairs bacterial growth [45]. In PAPI-1, *ndpA2* encodes an anti-histone-like nucleotide structuring protein (anti-H-NS), and *tprA* encodes a transcriptional regulator. H-NS, a chromosomally encoded nucleoid structuring protein, suppresses ICE gene expression. NdpA2 alleviates this repression by binding H-NS, and TprA subsequently activates transcription of the backbone genes [46].

Collectively, studies on ICE*clc* demonstrate that ICE activation is tightly controlled by multiple regulatory layers. While PsICEs share partial homology with their backbone, gene composition differs significantly, suggesting the involvement of additional or alternative regulatory modules. Actually, PsICE contains some backbone genes possibly related to ICE regulation. Some of these genes appear homologous to those found in PAPI-1. Additionally, as discussed in Section 4, PsICEs also carry additional backbone gene clusters, such as the *pil* gene cluster and the conserved DNA processing gene cluster, which may contribute to ICE specific regulation.

Several PsICEs have been experimentally shown to excise and transfer in vitro [25,26,27,28]. In *P. syringae* pv. *phaseolicola* 1302A, excision of PPHGI-1 is triggered by hydrogen sulfide, a plant-derived signal molecule [47]. This suggests that environmental and host-derived stresses may serve as cues for ICE activation in PsICE. However, the molecular mechanisms by which such signals are perceived and transduced remain unclear. To elucidate the roles of PsICEs in bacterial adaptation and plant interaction, it is crucial to further investigate the regulatory circuits and dynamics of PsICE mobilization, both *in vitro* and *in planta*.

## 6. Role of Cargo Genes in *Pseudomonas syringae*

In plant-pathogenic bacteria, ICEs have been identified in various species, including *P. syringae*, *Ralstonia solanacearum*, and *Xanthomonas campestris* [48,49]. These elements can carry cargo genes that potentially influence virulence, fitness, and antibiotic resistance [7,29,48,50]. The integration of pan-genomic analyses has been particularly informative in revealing the extensive variability of ICEs across bacterial strains. By comparing the genomes of multiple strains of a single species, researchers can identify backbone genes that are shared by all strains, as well as cargo genes that are present only in some strains [51]. These cargo genes contribute to the unique characteristics of individual strains, including their virulence, host specificity, and resistance to environmental stress. In *P. syringae*, for example, ICEs often harbor genes that influence pathogenicity and resistance to environmental challenges, further enhancing the adaptability and survival of the pathogen [48]. Pan-genomic studies have been instrumental in identifying novel ICEs that are associated with virulence, antibiotic resistance, and other adaptive traits, thus expanding our understanding of bacterial evolution and pathogen adaptation. The diversity in cargo genes across different strains is a key factor in the pathogen’s ability to adapt to new hosts, resist antibiotics, and survive in various environmental niches, further emphasizing the role of ICEs in bacterial evolution and adaptation.

*P. syringae* is a diverse bacterial species complex that causes significant agricultural losses by infecting various plants [52]. *P. syringae* employs multiple virulence strategies, including immune suppression and water soaking [52]. Genomic studies have revealed high genetic diversity and recombination rates within the species complex [53]. Recent genome sequencing has identified numerous virulence genes in *P. syringae* strains, including those related to biofilm formation, motility, and secretion systems [54]. Understanding these virulence mechanisms is crucial for developing effective control strategies in agriculture [54]. *P. syringae* and related bacterial species have been classified into 13 phylogroups (PGs) through multilocus sequence analysis (MLSA) [52]. These PGs are further grouped into two major clades: seven late-branching canonical lineages (PGs 1–6 and 10) and six early-branching noncanonical lineages (PGs 7–9 and 11–13) [52]. Notably, all plant-pathogenic *Pseudomonas* species fall within the late-branching canonical group [52]. In this section, we introduced several ICEs in pathogenic *P. syringae*.

**Figure 4:**
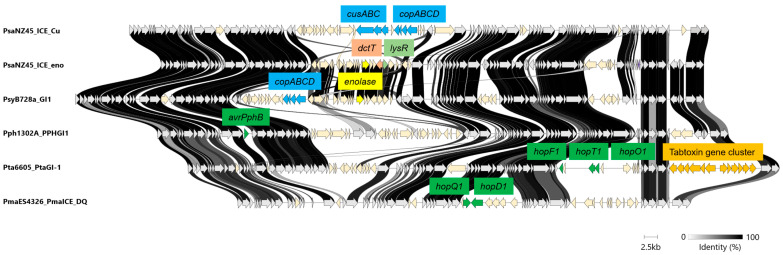
Structures of ICE in plant-pathogenic *Pseudomonas syringae*. ICEs in *P. syringae* pv. *actinideae* (PsaNZ45_ICE_Cu and PsaNZ45_ICE_eno), *P. syringae* pv. *syringae* B728a (PsyB728a_GI1), *P. syringae* pv. *phaseolicola* (Pph1302A_PPHGI1), *P. syringae* pv. *tabaci* 6605 (Pta6605_PtaGI-1) and *P. syringae* pv. *maculicola* ES4326 (PmaES4326_PmaICE_DQ) were compared. The arrows with light gray indicate the core genes. The arrows with light yellow indicate cargo genes. The green arrows represent the type III effector genes. The blue arrows represent the gene involved in copper resistance (*cusABC* and *copABCD*). The orange arrows represent the tabtoxin gene cluster. The yellow arrows represent the enolase gene. The orchid pink arrow represents the gene encoding transporter of dicarboxylic acids (DctT). The orchid green arrow represents the gene encoding the transcriptional factor LysR. The identity of each gene is shown as a degree of darkness.

*P. syringae* pv. *actinidiae* (PG1), the causative agent of bacterial canker in kiwifruit, has emerged as a significant global pathogen. Particularly, *P. syringae* pv. *actinidiae* biovar 3 is highly virulent and causes serious damage to kiwifruits worldwide [55]. A key factor in the evolution of *P. syringae* pv. *actinidiae* is the acquisition of resistance traits through HGT, particularly the acquisition of copper resistance. Studies have shown that strains of *P. syringae* pv. *actinidiae*, which were initially sensitive to copper-based treatments, have evolved copper resistance through the uptake of ICEs and plasmids [26]. Notably, *P. syringae* pv. *actinidiae* isolates from New Zealand, where copper-based sprays have been widely used, have been found to harbor mosaic structures in their ICEs, which include genes for copper resistance such as the *cusABC* and *copABCD* systems [26] (Figure 4). The *copABCD* operon is also identified in ICEs of another phylogroup *P. syringae* pv. *syringae* B728a (PG2) (Figure 4) [56]. The continuous genetic exchange mediated by ICEs highlights the pathogen’s dynamic adaptation processes, contributing to the emergence of more virulent and copper-resistant strains. Furthermore, Hemara et al. [57] explores the genomic evolution of *P. syringae* pv. *actinidiae* biovar 3 in New Zealand kiwifruit orchards. By sequencing over 500 isolates collected between 2010 and 2022, the study identified the loss of the effector gene *hopF1c*, which appears to be mediated by the movement of ICEs introducing copper resistance. The study found that *hopF1c* loss variants had similar in planta growth to wild-type *P. syringae* pv. *actinidiae* biovar 3, but a lab-generated ∆*hopF1c* strain exhibited a competitive advantage over the wild type on select hosts. Surveillance efforts confirmed that *hopF1c* loss variants remain limited in spread, and they are unlikely to cause more severe disease than the existing *P. syringae* pv. *actinidiae* biovar *3* population. The study highlights the importance of continued genomic biosurveillance to detect and manage emerging variants that could impact kiwifruit production.

A notable example of host metabolic adaptation via horizontal gene transfer in *P. syringae* pv. *actinidiae* is the widespread dissemination of a 16 kb mobile genetic element, Tn*6212*, which represents one of the most frequently integrated and highly conserved cargo gene clusters within PsICEs [29]. Tn*6212* encodes several cargo genes including a transporter of dicarboxylic acids (DctT) predicted to import tricarboxylic acid (TCA) cycle intermediates, a glycolytic enzyme enolase, and a LysR-type transcriptional regulator (Figure 4). Functional studies have demonstrated that Tn*6212* significantly enhances bacterial fitness under conditions enriched with TCA intermediates, such as those encountered in the plant apoplast, by mediating global transcriptional reprogramming of chromosomal genes potentially through mechanisms involving the RNA degradosome. This metabolic plasticity conferred by Tn*6212* suggests that PsICEs contribute to the fine-tuning of bacterial physiology in response to host-derived carbon sources, thereby revealing a previously underappreciated role of ICEs beyond the classical functions associated with virulence and antimicrobial resistance.

In *P. syringae* pv. *phaseolicola* (PG3), the genomic island PPHGI-1 carries the effector gene *avrPphB*, which triggers host resistance responses [25] (Figure 4). The genomic region containing *avrPphB* is located within a tRNA gene locus and is subject to excision, which can result in changes in virulence and host specificity. Strain RJ3, a variant of race 4, exhibits an extended host range in bean and soybean cultivars due to the absence of *avrPphB*, caused by the excision of a 40 kb chromosomal region [58]. This excision leads to a phenotype that resembles race 2, characterized by the loss of the avirulence gene but not a reduction in overall pathogenicity. Upon excision from the chromosome, PPHGI-1 undergoes supercoiling, suppressing *avrPphB* expression and preventing activation of host defenses. This excision process is strongly influenced by the bacterial population density and the presence of host resistance factors. In particular, passage of *P. syringae* through resistant bean plants increases ICE excision frequency, emphasizing the role of in planta stress in promoting genomic plasticity [59]. This regulatory mechanism underscores the dynamic role of GIs in modulating virulence gene expression in response to environmental or host cues. Furthermore, the topoisomerase encoded by the island, TopB3, stabilizes PPHGI-1, enhancing bacterial adaptability and virulence [47]. The excision event, mediated by homologous recombination between tRNA gene sequences, results in the re-formation of functional tRNA genes and a loss of the region carrying *avrPphB* [59].

In *P. syringae* pv. *tabaci* 6605 (PG3), the pathogenicity of the bacterium is significantly influenced by the presence of two genomic islands, *Pta*GI-1 and *Pta*GI-2 [28]. *Pta*GI-1, a pathogenicity island, carries a tabtoxin biosynthetic gene cluster and three type III secretion system effector genes, including *hopF1*, *hopT1*, and *hopO1* (Figure 4). These virulence factors enable the bacterium to cause severe disease symptoms in tobacco plants. In contrast, *Pta*GI-2 does not contribute to its virulence. The dynamic nature of these genomic islands, which can excise and circularize, further enhances the adaptability of *P. syringae* by facilitating the transfer and regulation of virulence genes. The loss of *Pta*GI-1 in mutant strains of *P. syringae* pv. *tabaci* resulted in a marked reduction in disease symptoms, confirming the critical role of tabtoxin in the pathogenesis of tobacco wildfire disease. These findings underscore the importance of genomic islands in the evolution and virulence of plant pathogens, illustrating how GIs facilitate the acquisition of phytotoxins and other virulence factors that enhance bacterial fitness and disease progression [28]. The *in silico* analysis has revealed that the tabtoxin biosynthetic gene cluster is indeed located within a genomic island, further supporting the concept that such islands play a crucial role in the acquisition and regulation of virulence factors in *P. syringae* [28].

In *P. syringae* pv. *maculicola* (also known as *P. cannabina* pv. *alisalensis*), ES4326 (PG5) and two ICEs, PmaICE-DQ and PmaICE-ABAO, are located in tandem [27]. PmaICE-DQ contains type III secretion system effector genes, *hopQ* and *hopD* (Fig. 4). The type III secretion system effectors HopD and HopQ are nearly identical and located in the same genomic region between *clpB* and *hopR* in *P. syringae* pv. *tomato* DC3000 and *P. syringae* pv. *maculicola* ES4326. This region forms a genomic island (~70 kb) corresponding to an ICE in *P. syringae* pv. *maculicola* ES4326. In *P. syringae* pv. *tomato* DC3000, the region appears to be a degraded ICE, with disrupted mobility genes. The evidence suggests that an ancestral ICE carrying *hopQ* and *hopD* was integrated and later immobilized in *Pto* DC3000 due to genetic decay. The loss of PmaICE-DQ did gain disease compatibility with *Nicotiana benthamiana* in a manner similar to the gain-of-compatibility phenotypes previously observed for *P. syringae* pv. *tomato* DC3000 *hopQ1-1* mutants [60]. Baltrus et al. [27] highlighted that under selective pressure from host immune responses, *P. syringae* strains can rapidly lose effector genes, allowing them to evade host defenses while maintaining the ability to infect new hosts. The excision of type III secretion system effector genes, coupled with the mobility of these genomic islands, enables *P. syringae* to modulate its virulence in a highly adaptive and context-dependent manner, ensuring its survival in the face of host immune responses. Although several studies implied the importance of cargo genes in ICEs, further studies are needed to understand the role of these genes especially in virulence.

## 7. Role of Genomic Island in Other Plant-Pathogenic Bacteria

GIs are key contributors to the virulence and adaptability of various plant-pathogenic bacteria, not just *P. syringae* but also other important plant pathogens such as *Pectobacterium atrosepticum*. GIs enable bacteria to acquire new traits that facilitate infection, host adaptation, and environmental persistence. In the case of *P. atrosepticum*, approximately 33% of the genes are unique to plant pathogens, with specific genes involved in nitrogen fixation and opine catabolism, highlighting the bacterial ability to adapt to various plant environments. The genome also contains horizontally acquired gene clusters associated with pathogenicity, such as those for type IV secretion and polyketide phytotoxin synthesis [61]. These findings underscore the role of HGT in expanding the virulence potential of *P. atrosepticum*, enabling rapid adaptation to different plant environments and the spread of virulence factors across bacterial populations.

In addition to metabolic and resistance-related genes, several pathogenicity islands in *P. atrosepticum* have been linked to virulence. Notably, one of the pathogenicity islands, HAI2, is responsible for the synthesis of coronafacic acid (CFA), a key virulence factor that facilitates the infection of potato plants. HAI8, associated with the type III secretion system, also plays a vital role in virulence [62]. The involvement of GIs in the regulation of virulence highlights their central role in the pathogenesis of *P. atrosepticum*, influencing toxin synthesis and immune evasion mechanisms.

The role of topoisomerase IIIb (PbTopo IIIb) in the excision of HAI2 further emphasizes the complex regulation of GIs and their impact on virulence. PbTopo IIIb is essential for the stable maintenance of HAI2, and its inactivation leads to significant excision of the island, resulting in reduced fitness and decreased virulence on potato plants [62]. The study suggests that PbTopo IIIb plays a critical role in maintaining the stability of pathogenicity islands, and its disruption leads to hyper-excision of HAI2, altering the transcription of key virulence genes such as *cfa6* and *cfa7*, which are involved in CFA biosynthesis. These findings underscore the importance of topoisomerase IIIb in regulating pathogenicity islands and controlling bacterial virulence. Furthermore, recent studies employing microarray comparative genomic hybridization (aCGH) have expanded our understanding of GIs in plant-pathogenic enterobacteria. This technique has been instrumental in identifying GIs linked to pathogenicity, host range, and environmental persistence in species such as *P. atrosepticum*, *P. carotovorum*, and *Dickeya* spp. The aCGH analysis reveals strain-specific phenotypes associated with phytotoxin synthesis, multidrug resistance, and nitrogen fixation, crucial factors for the pathogen’s ability to adapt and cause disease. Notably, the study found that a significant portion of the *P. atrosepticum* genome has no orthologs in *Dickeya* spp. or *P. carotovorum*, pointing to the acquisition of new genes via HGT [63]. This highlights the plasticity of bacterial genomes and the pivotal role of the “accessory genome” in shaping pathogen behavior, environmental survival, and pathogenicity.

Similarly, *Ralstonia solanacearum*, the causative agent of bacterial wilt, also harbors numerous GIs that contribute to its virulence and adaptability. Gonçalves et al. [49] identified 12 ICEs and 31 genomic islands in *Ralstonia* genomes, with some elements accounting for up to 5% of the host genome. These elements carry genes involved in virulence, stress response, and metabolic processes essential for host adaptation [49]. In a comprehensive genomic analysis of 300 plant-pathogenic bacteria, de Assis et al. [48] highlighted the broad distribution of ICEs across species such as *P. syringae*, *R. solanacearum*, and *X. campestris*. The study found that 78 distinct ICEs were identified, many of which carried cargo genes linked to virulence and fitness. These genes, including those involved in T3SS, hydrolytic enzymes, and metal resistance, underline the contribution of ICEs to the adaptability and pathogenicity of plant-pathogenic bacteria [48]. These studies demonstrated that the presence of ICEs in bacterial genomes directly impacts their capacity to infect plants, providing them with an evolutionary advantage in diverse ecological niches.

In *Erwinia amylovora*, the causative agent of fire blight, the *hrp* pathogenicity island is central to its virulence. The *hrp* pathogenicity island encodes key components of the T3SS, facilitating the injection of effector proteins into host plant cells and modulating plant immune responses. The identification of novel genes, such as *hsvA*, *hsvB*, and *hsvC*, further emphasizes the importance of GIs in the infection process, as these genes are required for systemic infection in apple trees [64]. Comparative analysis of the *hrp* pathogenicity island in *E. amylovora* with other plant pathogens highlights conserved and unique elements that contribute to the pathogen’s ability to infect a broad range of plant species.

In addition to ICEs, prophages and plasmids are common MGEs that contribute to bacterial adaptation and virulence [65]. In *Xylella fastidiosa*, a destructive plant pathogen affecting crops like olive and citrus trees, prophages are abundant and play a significant role in pathogenicity. This bacterium relies on genes encoding toxins, enzymes, adhesins, and cell–cell aggregation proteins to cause disease. A study analyzing 94 *X. fastidiosa* genomes found that while insertion sequences (ISs) are modestly distributed, prophages are more widespread and contribute to numerous pathogenicity genes. Phylogenetic analysis suggests that these prophages, recently acquired by the pathogen, can rearrange genes and create strains with new pathogenic capabilities. The study also demonstrated that MGEs, including prophages, are strongly associated with virulence genes, underscoring their role in pathogenesis [66].

Similarly, prophages and GIs in *Pseudomonas fluorescens* Pf-5 carry niche-specific genes that may influence bacterial survival in various environments. In particular, prophages in *P. fluorescens* Pf-5, such as F-pyocin-like prophage 01, contribute to the bacteria’s ability to thrive in the rhizosphere, providing protection against plant pathogens and enhancing survival in diverse ecological niches [67]. These prophages, which are integrated into the bacterial genome, carry genes involved in bacteriocin production and lysis of competitor bacteria, further promoting the survival and competitiveness of *P. fluorescens* in complex environments.

## 8. Future Research Direction

GIs including ICEs, prophages, and integrative plasmids drive bacterial genome plasticity, antibiotic resistance, and virulence dissemination [68,69]. With the rapid growth of sequenced genomes, future research must integrate mechanistic, ecological, and translational approaches to both harness beneficial traits and curb the spread of harmful determinants.

Elucidating GI mobilization and regulation at the molecular level is a priority. High-resolution structural biology and biochemical assays of integrases, excisionases, and relaxases will identify critical residues and conformational changes governing recombination [15]. Concurrently, transcriptomic and reporter-gene studies under abiotic stresses (e.g., nutrient limitation, UV exposure) and host-derived cues (e.g., phytochemicals, reactive oxygen species) will reveal environmental triggers for element activation and horizontal gene transfer. Recent functional analyses of prophages in *P. syringae* demonstrate that prophage-mediated mobilization is not only biochemically inducible but also ecologically relevant. In particular, prophages carrying the type III secretion system effector gene *hopAR1* excise, circularize, and transfer on cherry leaf surfaces when exposed to UV or mitomycin C treatments, confirming that environmental stimuli encountered in the phyllosphere directly trigger GI activation. Moreover, prophage induction was abolished by mutating the integrase gene, underscoring the mechanistic role of site-specific recombinases in horizontal gene transfer [70]. This phyllosphere-based transfer highlights the need to dissect GI regulation both in vitro and in planta. Molecular dissection of attachment sites and integrase specificity, combined with live-cell reporter assays in planta, will deepen our understanding of how phage–host interactions and environmental cues converge to control GI mobilization.

Delving deeper into horizontal gene transfer mechanisms including conjugation, transduction, and transformation will enable the development of targeted interventions for plant pathogen control. High-throughput screening of chemical libraries can identify small-molecule inhibitors that selectively block key GI-associated enzymes, such as relaxases and conjugative ATPases, without perturbing essential bacterial functions [71]. In parallel, CRISPR-based platforms including Mobile-CRISPRi and other Cas effectors can offer precise in situ silencing of core mobility and virulence-related genes, thereby restoring fungicide sensitivity and attenuating bacterial pathogenicity in planta [72,73]. By integrating structural insights with functional genomics, these approaches promise to thwart the emergence and dissemination of new virulence or resistance traits in agricultural settings [74,75].

Investigating the spatiotemporal dynamics of element-encoded virulence factors such as effectors, toxins, and secretion systems during infection will deepen our understanding of host–pathogen interactions. Single-cell RNA-seq, fluorescent reporter strains, and dual RNA-seq enable simultaneous profiling of host and pathogen transcriptomes, pinpointing when and where GIs deploy these virulence determinants [76,77]. Comparative infection assays across diverse plant hosts and environments will clarify how variation in MGE content influences host specificity, colonization efficiency, and disease severity.

Recent longitudinal biosurveillance of *P. syringae* pv. *actinidiae* in New Zealand kiwifruit orchards revealed that intensive agricultural practices, such as widespread copper spray application and monoculture cultivation, exert strong selection pressures that accelerate bacterial genome adaptation [57]. These practices were associated with the replacement of native ICEs by copper-resistance ICEs, as well as the repeated loss of effector genes such as *hopF1c*. Strikingly, these effector deletions were often mediated by ICE excision events, illustrating how GIs are key conduits for adaptation in agroecosystems. This underscores a critical need to study how anthropogenic environments, characterized by chemical inputs and genetic uniformity, select for GI-mediated virulence and resistance evolution. Future GI research must therefore expand beyond molecular mechanisms to encompass the ecological and sociotechnical dimensions of GI evolution in managed ecosystems. Such an integrative approach will inform sustainable disease control strategies that anticipate, rather than merely respond to, pathogen adaptation.

To effectively anticipate such GI-driven evolutionary shifts at the population level, it is essential to implement comprehensive genomic surveillance of field isolates. Whole-genome and metagenome sequencing, combined with advanced bioinformatics pipelines, enables the detection of emerging GIs that harbor virulence or resistance determinants and allows for real-time tracking of their dissemination across agricultural landscapes [78,79]. Integrating sequencing data with GIS mapping and AI-driven predictive models enables forecasting of outbreak hotspots and transmission pathways [80,81]. Addressing challenges in data standardization, analytical complexity, and resource availability through open data sharing, capacity building, and international collaboration will be essential for global implementation of effective genomic surveillance systems [82]. Finally, a comprehensive, publicly accessible database is needed to catalog GIs across all sequenced bacterial genomes. By integrating tools like IslandViewer [83], ICEberg [16,84], PHASTER [85], Design-Island [86], CG-MJSD [87], and mobileOG-db [88] with rich metadata on host taxonomy, ecological origin, and leveraging scalable cloud infrastructure and Application Programming Interfaces, this platform enables researchers to search, compare, and visualize the complete repertoires of GIs, thereby accelerating both fundamental discovery and practical disease control strategies.

## 9. Conclusions

Genomic islands and related mobile genetic elements represent pivotal engines of adaptability and virulence in plant-pathogenic bacteria. By shuttling clusters of functionally linked genes including toxins, secretion systems, resistance determinants, and metabolic pathways, these elements facilitate rapid phenotypic shifts that underpin host specialization, immune evasion, and the emergence of novel epidemic strains. The modular architecture and lifecycle dynamics of ICEs, prophages, and pathogenicity islands underscore the intricacy of regulatory circuits that govern excision, transfer, and integration, often operating through bistable cell-fate decisions and environmental cues.

Advances in high-throughput sequencing, coupled with computational platforms integrating resources such as IslandViewer, ICEberg, PHASTER, Design-Island, CG-MJSD, and mobileOG-db have dramatically expanded our ability to catalog and compare MGEs across diverse strains and ecological niches. However, translating descriptive inventories into mechanistic understanding requires unified standards for metadata annotation, robust experimental systems to probe GI regulation *in planta*, and novel tools such as CRISPR-based modulation to dissect cargo gene function in real time.

Looking ahead, interdisciplinary efforts that combine structural biology, single-cell analyses, and ecological modeling will be essential to predict and preempt the trajectories of GI-driven pathogen evolution. Integrative databases, enriched with host taxonomy, environmental context, and gene cargo profiles, will empower the development of targeted interventions ranging from phage-derived antimicrobials to anti-conjugation inhibitors that disrupt horizontal gene transfer pathways. Ultimately, a deep mechanistic grasp of genomic islands and their regulatory networks will pave the way toward sustainable disease control strategies, mitigating the threat of rapid adaptation in agricultural ecosystems.

## Data Availability

No new data were created or analyzed in this study.

## Abbreviations

The following abbreviations are used in this manuscript:

HGT	Horizontal gene transfer
GI	Genomic island
ICE	Integrative and conjugative element
RDF	Recombination directionality factor
ssDNA	Single-strand DNA
T4CP	Type IV coupling protein
T4SS	Type IV secretion system
Tc cell	Transfer-competent cell
MLSA	Multilocus sequence analysis
H-NS	Histone-like nucleotide structuring protein
PG	Phylogroup
TCA	Tricarboxylic acid
CFA	Coronafacic acid
T3SS	Type III secretion system
aCGH	array comparative genomic hybridization
